# Z-DNA-associated genomic instability in the human pangenome

**DOI:** 10.21203/rs.3.rs-10742736/v1

**Published:** 2026-09-08

**Authors:** Ilias Georgakopoulos-Soares, Georgios Megalovasilis, Eleftherios Bochalis, Dionysios Chartoumpekis, Karen Vasquez

**Affiliations:** The University of Texas at Austin; The University of Texas at Austin; The University of Texas at Austin; University of Texas at Austin; University of Texas at Austin

## Abstract

Z-DNA is a non-canonical, left-handed nucleic acid conformation implicated in gene regulation and genome instability. Long-read sequencing and telomere-to-telomere genome assemblies now enable analysis of repetitive regions that were absent or incompletely represented in earlier human references. Leveraging T2T-CHM13 and 464 haplotype-resolved Human Pangenome Reference Consortium assemblies, we mapped predicted Z-DNA-forming sequences and characterized their genomic and mutational landscape. Z-DNA density was broadly similar across human haplotypes and superpopulations but differed substantially between T2T-CHM13 and GRCh38 in several repetitive genomic compartments, including acrocentric loci. Across more than 52 million HPRC variants, predicted Z-DNA-forming loci showed elevated mutation density relative to length-, GC-, and CpG/GpC-matched controls across most mutation classes, with the strongest associations observed for small and intermediate insertions, deletions, and complex insertion- and deletion-like events. Enrichment was concentrated near predicted B-Z junctions and varied by genomic context, with the strongest effects in genic and euchromatic compartments. Similar mutation-class associations were observed in more than 250,000 de novo mutations. Together, these analyses define the mutation-class, spatial, and genomic-context landscape associated with predicted Z-DNA-forming sequences across the human pangenome.

## Introduction

Z-DNA is a left-handed DNA helix that represents an alternative conformation to the canonical right-handed B-DNA (1). It is characterized by a zig-zag phosphate backbone and alternating *syn/anti* base conformations. Formation of Z-DNA is favored by specific sequence contexts, such as alternating purine-pyrimidine tracts of (CG)n repeats (2), whereas AT repeats are disfavored (3). Z-DNA formation is influenced by several other factors, including CpG methylation, ionic strength, torsional stress, and negative supercoiling, which either promote or hinder the transition from B-DNA to the left-handed Z conformation (4).

Z-DNA has been implicated in multiple cellular processes (5, 6). For example, Z-DNA is enriched near transcription start sites, can modulate *cis*-regulatory activity, and has functional roles in chromatin remodelling (7-12). Previous studies have also provided evidence for its role as a nucleosome-boundary element and a regulator of local chromatin accessibility (13, 14). Proteins with Z-nucleic acid-binding domains, such as ADAR1 and ZBP1, recognize and stabilize Z-DNA and Z-RNA structures (15), which has been linked to innate immune signaling, antiviral responses, and programmed cell death (8, 16, 17).

Beyond regulatory roles, Z-DNA-forming sequences have been associated with genomic instability in both experimental and computational studies. Z-DNA-forming sequences can stimulate DNA strand breaks and large deletions in mammalian cells (18, 19), while genome-wide analyses have linked non-B DNA motifs, including Z-DNA-forming motifs, to elevated nucleotide substitution frequencies and regional variation in human polymorphism and interspecies divergence (20, 21). More recently, genome-wide prediction of Z-forming loci has associated these sequences with regulatory and disease-associated genetic variation (22, 23). Together, these studies provide substantial prior evidence linking Z-DNA-forming potential with genomic variation and instability. However, the mutation-class spectrum associated with these loci and its distribution across repetitive regions resolved by complete human genome and pangenome assemblies remain incompletely characterized.

Previous analyses were performed using pre-T2T reference genomes or, in some cases, deliberately restricted to non-repetitive or otherwise reliably interrogated genomic regions. The advent of telomere-to-telomere (T2T) genome assemblies (24) and panels of haplotype-resolved human genomes spanning diverse ancestries (25), enabled by long-read sequencing technologies, now provides access to repetitive and structurally complex regions that were absent, collapsed, or incompletely represented in earlier references. These include centromeric and pericentromeric satellite arrays, telomeric and subtelomeric regions, the rDNA-containing short arms of the acrocentric chromosomes, and segmental duplications (24).

Several computational tools have been employed for the detection of Z-DNA-forming sequences (22, 26-29). Guided by experimental data, we recently published ZSeeker, a fast and scalable computational tool that predicts Z-DNA-forming sequences genome-wide (30). ZSeeker has been validated against *in vivo*, *in vitro*, molecular, biochemical, biophysical studies, and structural datasets. Here, we used ZSeeker to characterize the distribution of predicted Z-DNA-forming sequences in T2T-CHM13 and across 464 haplotype-resolved HPRC assemblies, and to determine how their association with human variation depends on mutation class, variant size, local position, genomic context, and allele frequency

We found that predicted Z-DNA-forming sequence density was 214 bp/Mb higher in T2T-CHM13 than in GRCh38, with prominent differences in repetitive regions that are more completely represented in the T2T assembly. Across more than 52 million HPRC variants, most mutation classes showed elevated density near predicted Z-DNA-forming sequences relative to length-, GC-, and CpG/GpC-matched controls, with the strongest associations observed for small and intermediate indels and complex insertion- and deletion-like events. This enrichment was spatially concentrated near predicted B-Z junctions and varied markedly by genomic context, with the strongest effects in genic and euchromatic compartments. Similar mutation-class associations were observed in an independent set of more than 250,000 de novo mutations, arguing against population-level selection as the sole explanation for the observed pattern. Together, these analyses define the mutation-class, spatial, and genomic-context landscape associated with predicted Z-DNA-forming sequences across the human pangenome.

## Results

### Characterization of Z-DNA predictions across human haplotypes

Using the T2T (24) reference human genome from the CHM13hTERT cell line (T2T-CHM13), as well as the 464 T2T human haplotypes provided by the second release of the HPRC (25), we examined the distribution and population-wide conservation of Z-DNA-forming sequences across human chromosomes. The set of haplotypes comprised allelic profiles from diverse ancestries, across five super-populations: African (AFR, *n* = 140); East Asian (EAS, *n* = 102); American (AMR, *n* = 88); South Asian (SAS, *n* = 72); and European (EUR, *n* = 62).

Using the approach outlined in Fig. 1a, we found a Z-DNA density of 4,897 bp/Mb for T2T-CHM13, compared to 4,683 bp/Mb for GRCh38 and a mean Z-DNA density of 4,837 bp/Mb across the 464 HPRC haplotypes (Fig. 1b). The lower Z-DNA density in GRCh38 likely reflects, at least in part, repetitive regions that are absent or incompletely represented in this reference compared with T2T-CHM13. Z-DNA density was stable across superpopulations (Fig. 1c), whereas the number of haplotype-specific Z-DNA predicted sequences varied substantially. AFR assemblies exhibited the highest counts of haplotype-specific sequences (up to 2,035), while AMR and EUR haplotypes generally showed lower counts (Fig. 1d). CHM13 contained comparatively few haplotype-specific Z-DNA sequences (n = 601). At the same time, GRCh38 featured a higher number (n = 1,848), consistent with its multi-donor assembly composition.

ZSeeker provided a Z-DNA score metric for each prediction, quantifying the propensity of a sequence to adopt the Z-conformation (30). Comparing the two reference genomes and the five superpopulations (Fig. 1e), we observed highly similar score distributions across all groups, with nearly identical distributions. Predicted Z-DNA sequence lengths followed the same pattern (**Suppl. Figure 1a**). When stratifying Z-DNA density by chromosome (Fig. 1f), we observed chromosome-specific differences in median density that were strongly associated with chromosomal GC content (Spearman *ρ* = 0.74, *p* = 7.035 × 10^−5^) (**Suppl. Figure 1b**). Consistent with previous genome-wide Z-DNA prediction maps (22), acrocentric chromosomes displayed prominent Z-DNA signal. Complete T2T resolution allowed this signal to be quantified across rDNA- and satellite-rich short-arm sequence that is absent or incompletely represented in GRCh38. Chromosomes 13 and 21 showed the largest increases in Z-DNA density relative to GRCh38, by 2,779 and 4,589 bp/Mb, respectively (Fig. 1f; **Supplementary Table 1**).

We also asked whether, in trio-phased assemblies, the autosomes exhibit a consistent paternal-maternal shift in Z-DNA signal that would indicate a parent-of-origin effect (**Suppl. Figure 1c**). Across 128 individuals, the median paternal-maternal difference was + 8.6 bp/Mb, with no significant paired difference observed between haplotypes (Wilcoxon signed-rank *p* = 0.082), indicating no systematic parent-of-origin bias in autosomal Z-DNA density. These results indicate broadly similar genome-wide Z-DNA density across the examined haplotypes, without detectable systematic ancestry- or parent-of-origin-associated differences.

### Z-DNA is enriched at transcriptionally active genomic loci in the CHM13 human genome

Genome-wide ideogram visualization of T2T-CHM13 (Fig. 2a) showed a non-uniform distribution of predicted Z-DNA-forming sequences, including prominent signal at the rDNA-containing short arms of acrocentric chromosomes, consistent with previous computational analyses and prior evidence for Z-DNA-forming potential in rDNA (22, 30, 31). We also observed discrete Z-DNA enrichment peaks at additional loci, including tRNA clusters, SST1 satellite regions, and D4Z4 repeat arrays (Fig. 2a). Quantification of Z-DNA density across cytoband stain categories showed that euchromatic cytobands (gneg and gpos25) exhibited higher density than heterochromatic bands (gpos75 and gpos100) (median 4.68 vs. 3.45 bp/kb; Mann-Whitney U, *q* = 2.9 × 10^−24^; Cliff's *δ* = 0.52; Fig. 2b). Centromeric regions (acen) were almost entirely depleted of Z-DNA relative to non-centromeric cytobands (median 0.0 vs. 4.1 bp/kb; *q* = 7.2 × 10^−31^; Cliff's *δ* = −0.999). The highest densities were observed in stalk regions, which correspond to nucleolar organizer/rDNA loci (median 90.8 bp/kb; *q* = 1.1 × 10^−4^ vs. all other cytobands). Subsequent analysis of centromeric satellite regions confirmed and extended these results (Fig. 2c). Examining sex chromosome-specific regions (**Suppl. Figure 1d**) Z-DNA density varied, with pseudoautosomal regions (PAR) showing the highest median density (12,828 bp/Mb; n = 4), followed by ampliconic (median 5,077 bp/Mb; n = 7) and X-degenerate regions (median 4,112 bp/Mb; n = 8). Satellite, centromeric, and heterochromatic regions exhibited lower densities, consistent with the depletion pattern observed earlier.

Next, we quantified Z-DNA enrichment across 34 genomic annotation categories relative to the genome-wide Z-DNA density (Fig. 2d-e). Consistent with the known abundance of non-canonical structure-forming sequences in rDNA (31), rDNA arrays showed the strongest enrichment among the 34 annotation categories examined (18.84-fold), followed by tRNA arrays (4.71-fold) and CenSat repeats (2.17-fold; Fig. 2d-e; **Supp. Table 2**). Within CenSat repeats, HSAT1A (no detectable Z-DNA), HSAT2, HSAT3, and HOR and AHOR were depleted (< 0.01-0.15-fold), whereas BSAT, GSAT, and CT regions showed moderate to minimal depletion (0.76-0.95-fold). Among interspersed repeat classes, all major transposable element families (LTR, DNA transposons, SINEs, and LINEs) were depleted (0.16-0.69-fold), whereas simple repeats (11.29-fold) and retroposons (2.92-fold) were strongly enriched. For gene biotypes, miRNA genes showed the highest enrichment (4.29-fold), while protein-coding genes (1.12-fold) and pseudogenes (1.19-fold) were also enriched. Among gene substructures, coding sequences (6.01-fold) and exons (3.87-fold) were strongly enriched, gene bodies were near baseline (1.06-fold), and introns were slightly depleted (0.94-fold).

We conclude that Z-DNA is enriched in euchromatic, transcription-associated loci, largely excluded from major heterochromatic repeat compartments, and highly enriched at transcriptionally active loci and rDNA genes.

### Predicted Z-DNA-forming loci are associated with elevated mutation density across most mutation categories

To directly assess whether Z-DNA loci represent localized hotspots of genomic instability, we next examined mutation burden across haplotypes obtained from the deconstruction of the HPRC pangenome graph. We classified 52 million mutations into 21 mutation categories (based on their type, size, and breakpoints in the case of large variants) from single-nucleotide variants (73.5% of events) to complex indels (3.2%; **Suppl. Figure 1e**). To determine whether Z-DNA-forming sequences are preferential sites of mutation, we examined mutational enrichment across variant categories using two complementary approaches.

To test whether Z-DNA-forming sequences are associated with elevated mutagenesis, we computed the relative density of these variants within the vicinity of predicted Z-DNA intervals versus composition-matched control regions (Fig. 3a). Z-DNA loci displayed a pronounced enrichment peak centered on the mutation site, compared to composition-matched control regions, which remained largely flat and close to the normalized baseline (Fig. 3b). The majority of mutation categories showed significant enrichment near Z-DNA (Wilcoxon signed-rank test, *q* < 0.01 for 18 out of the 21 mutation categories). The strongest effects were observed for small- and medium-sized complex indels (Fig. 3b): complex deletions of 6–50 bp (9.8-fold), complex insertions of 6–50 bp (7.4-fold), and complex deletions of 1–5 bp (3.2-fold).

Beyond complex events, most simple deletions and insertions displayed Z-DNA enrichment (1.2- to 3.1-fold), as did multi-nucleotide variants and small substitutions (2.2-fold). SNVs exhibited a more modest enrichment (1.3-fold), but when all six substitution categories were examined, T > C transitions showed the strongest effect (1.45-fold), followed by T > G, T > A, and C > T (1.23-1.25-fold) (**Suppl. Figure 2a**). Conversions C > A and C > G displayed the weakest enrichment values (1.07-1.09-fold; **Suppl. Figure 2a**). Finally, a few categories showed no significant enrichment (**Suppl. Figure 2b**; *q* > 0.01), two representing larger structural variants: insertions and substitutions greater than 200 bp (0.98-1.20-fold) and 1 bp insertions (0.93-fold).

To determine how tightly the observed association was spatially localized relative to predicted Z-DNA intervals, we divided each Z-DNA sequence into length-normalized bins and compared mutation frequencies across the interval and its flanking regions with those of the matched control sequences using Z-DNA-centred metaprofiles (Fig. 3c). For the six representative mutation categories, log_2_(Z-DNA/Control) mutation frequency was highly elevated within the Z-DNA sequence, and specifically peaked near the predicted B-Z junctions and decayed toward baseline in the flanking regions. Complex deletions of 6–50 bp exhibited the strongest enrichment (37.9-fold), followed by complex insertions of 6–50 bp (28.0-fold), simple deletions of 6–50 bp (9.7-fold), simple insertions of 6–50 bp (6.0-fold), and MNVs (4.7-fold), while SNVs displayed modest enrichment (1.6-fold) and 1 bp insertions (**Suppl. Figure 2c**) featured a mild depletion (0.7-fold; 95% CI: 0.6–0.8). All comparisons were statistically significant on chromosome-level frequencies (one-sided Wilcoxon signed-rank test, *q* = 6 × 10^−8^). These results indicate that most mutation classes are preferentially clustered near predicted B-Z junctions.

### Z-DNA-associated mutation enrichment varies with variant size, Z-DNA score, and allele frequency

To test whether Z-DNA-associated mutagenesis depends on variant size, we determined Z-DNA enrichment within a window around mutation anchors. Windows of ± 10, ±25, and ± 50 bp were evaluated to assess the spatial concentration of the effect. Z-DNA enrichment levels were strongly linked to the variant size. The four indel and complex variant categories showed the strongest Z-DNA enrichment at intermediate event sizes. Complex deletion-like variants were the most enriched, peaking at 44.1-fold within ± 10 bp for the 11–20 bp length, followed by complex insertion-like variants (26.2-fold) and deletions (22.7-fold), both of which also peaked at the 11–20 bp length (Fig. 4a). Insertions showed the highest enrichment at 2 bp length (17.3-fold) but also featured a second, broader maximum at 51–100 bp length (16.8-fold; **Suppl. Figure 2d**). MNVs showed the highest enrichment (30.9-fold) at 11–20 bp. Across all categories, enrichment attenuated as the window widened, with the ± 25 and ± 50 bp curves falling consistently below ± 10 bp, indicating that the association is concentrated at the variant site rather than reflecting a broader regional effect.

We next asked whether Z-DNA-associated mutagenesis varies with predicted Z-DNA score. For this, we partitioned the predicted Z-DNA loci in T2T-CHM13 into three equal-count terciles of increasing Z-DNA score (G1 < G2 < G3), which also differed in Z-DNA tract length, with median lengths increasing from 30 bp in G1 to 44 bp in G2 and 61 bp in G3. We then recomputed mutation-anchored enrichment profiles separately for each score group and quantified each score group’s contribution to the total excess Z-DNA-associated signal. Across many mutation classes, particularly MNVs, small and intermediate indels and complex indels, and short substitutions, the lower- and intermediate-score groups carried most of the excess enrichment signal (Fig. 4b), whereas G3 showed significantly reduced signal relative to both G1 and G2 (chromosome-level pairwise Wilcoxon signed-rank tests, *q* < 0.05). The strongest examples were complex deletions of 6–50 bp, complex insertions of 6–50 bp, 6–50 bp deletions, 2–5 bp deletions, 6–50 bp insertions, MNVs, and 20–50 bp substitutions, for which G1 and/or G2 showed substantially higher enrichment than G3. SNVs showed a distinct monotonic pattern (Fig. 4c), with excess signal decreasing from G1 to G2 to G3 (mean relative density 1.70, 1.52, and 1.43, respectively; all pairwise *q* < 0.001). In contrast, larger-event classes showed weaker and less consistent score-dependent behavior, with some categories showing no significant pairwise differences and others showing higher enrichment in G3. Representative metaprofiles (Fig. 4c) illustrate these regimes: a sharp score-ordered central peak for SNVs, strong G1/G2-over-G3 separation for complex 6–50 bp deletions, and broader, less consistently ordered profiles for larger events such as complex deletions > 50 bp and substitutions > 200 bp. One possible explanation is that higher-scoring Z-DNA regions also tend to be longer, and very long predicted Z-DNA tracts may be less likely to form or maintain stable Z-DNA structures in vivo.

### Z-DNA is enriched around mutations in genic and euchromatic compartments

To examine differences in Z-DNA-associated genomic instability across genomic sub-compartments, we compared, for each mutation category and genomic feature, the odds that a variant lies within ± 10 bp of a Z-DNA interval versus a length- and composition-matched control interval using a discordant McNemar odds ratio (Fig. 4d). Features were tested independently, so a mutation could contribute to multiple genomic-feature cells.

Out of the 96 feature-mutation comparisons (Fig. 4d), 94 were statistically significant (*q* < 0.05): 71 showed enrichment of mutations near Z-DNA relative to matched controls (OR > 1), whereas 23 showed depletion (OR < 1). Enrichment was concentrated in genic compartments and LINEs, while depletion was concentrated in SINEs, RepeatMasker satellites, and centromeric satellites regions. Complex deletions showed the strongest associations in exons, introns, gene bodies, protein-coding genes, and lncRNAs (OR = 20.8–22.3), with lower but still marked enrichment in coding sequences and pseudogenes. Complex insertions were also strongly enriched across these genic features, particularly in lncRNAs, gene bodies, introns, protein-coding genes, and exons (OR = 11.3–16.7; *q* < 0.05). SNVs showed comparatively modest enrichment in genic regions (OR = 1.02–1.63), although stronger SNV enrichment was observed in LINEs (OR = 2.33). In contrast, SINEs and RepeatMasker satellites showed consistent depletion across all mutation classes (OR = 0.13–0.57), and CenSat regions were also depleted for most mutation classes (OR = 0.06–0.58), with complex deletions in CenSat being the only non-significant depletion-like comparison (OR = 0.94). CTs were a clear exception among CenSat annotations, showing significant Z-DNA-associated enrichment across all mutation classes (OR = 1.35–13.44).

We also assessed whether their Z-DNA association varied within-HPRC allele frequency, separately for coding and noncoding sequences. In noncoding sequences, the ± 5 bp Z-DNA/control peak ratio at SNV sites rose monotonically with allele frequency, from 1.23-fold for very rare alleles to 1.42-fold for those with a frequency above 5% (*q* = 0.002; Fig. 4e-f), and this positive trend was stable across allele-number thresholds (counts of haplotypes harboring info for a specific allele) from 10 to 400. Coding SNVs showed no significant trend. Higher-frequency SNVs are therefore more concentrated near Z-DNA in noncoding sequences specifically, highlighting a compartment-specific relationship.

Together, these results indicate that SNVs with higher within-HPRC allele frequencies are preferentially concentrated near Z-DNA-forming sequences in noncoding regions, whereas coding SNVs show no comparable frequency-dependent pattern. This suggests a compartment-specific relationship between Z-DNA and standing genetic variation, potentially reflecting differences in mutational processes, tolerance, and/or constraint between coding and noncoding sequences.

#### Z-DNA mutational enrichment is detectable in de novo mutations

Population variants reflect genetic variation that has persisted in human populations and has therefore been exposed to the effects of natural selection. To test whether Z-DNA-associated mutagenesis is detectable before selective forces can eliminate or retain variants, we performed the same analysis on 255,841 *de novo* WGS-derived mutations from denovo-db (32) (Fig. 5a) on the GRCh37 reference genome. *De novo* SNVs (*n* = 228,896; **Suppl. Figure 2e**) showed significant Z-DNA enrichment (1.1-fold; one-sided, *q* = 1.5 × 10^−4^), comparable to the population-level HPRC signal (1.3-fold). Simple deletions (*n* = 10,414; 2.0-fold) and insertions (*n* = 7,549; 2.2-fold) were enriched, and the strongest effects were again observed for complex events: complex deletion-like (*n* = 6,546; 2.4-fold) and complex insertion-like (*n* = 1,955; 1.9-fold) variants (all *q* values < 0.02). Across all categories, the matched control profiles remained close to the normalized baseline (Fig. 5b).

## Discussion

In this study, we mapped predicted Z-DNA-forming sequences across T2T-CHM13 and 464 haplotype-resolved HPRC assemblies and characterized their association with more than 52 million human variants. Mutation density was elevated near predicted Z-DNA-forming loci relative to length-, GC-, and CpG/GpC-matched controls across most mutation classes, with the strongest associations observed for small and intermediate indels and complex insertion- and deletion-like events. The association was highly localized, with enrichment concentrated within predicted Z-DNA-forming intervals and near predicted B-Z junctions, and varied substantially among genomic compartments. Similar class-level patterns were observed in an independent set of more than 250,000 de novo mutations. Together, these results extend previous evidence linking Z-DNA-forming sequences to genomic instability by defining the mutation-class, spatial, and genomic-context landscape of this association in complete and haplotype-resolved human genomes.

Previous studies have already established important relationships between non-B DNA-forming sequences and genomic variation. Genome-wide analyses of human polymorphism and human-orangutan divergence showed elevated substitution frequencies at non-B DNA motifs and identified Z-DNA motif coverage as a significant contributor to regional variation in divergence and diversity (21). More recently, Z-DNABERT-based analyses connected predicted and experimentally supported Z-forming loci with regulatory variation and disease-associated variants (22). Our results therefore do not establish the general mutagenic association of Z-DNA-forming sequences de novo. Rather, the use of T2T-CHM13 and the HPRC pangenome extends this framework to repetitive regions that were absent or incompletely represented in earlier reference assemblies and resolves the association across a broad spectrum of mutation classes. The higher Z-DNA density in T2T-CHM13 relative to GRCh38, particularly at rDNA- and satellite-rich acrocentric short arms, illustrates the contribution of these newly accessible genomic compartments. The pronounced rDNA enrichment is also consistent with previous evidence that human rDNA contains abundant non-canonical structure-forming sequences, including sequences capable of adopting the Z conformation (31).

Several experimentally supported mechanisms are compatible with the localized indel- and deletion-associated patterns observed here. Z-DNA-forming sequences can be recognized and processed by the MSH2-MSH3 mismatch-repair complex and ERCC1-XPF, generating strand breaks near structure-forming regions (19). In a distinct vertebrate model, alternative-structure-forming TG repeats within the stickleback Pitx1 enhancer produced strongly orientation-dependent chromosome fragility; in mammalian cells, the most mutagenic TG-repeat construct predominantly generated deletions exceeding 100 bp, with many deletion junctions containing microhomology and insertions consistent with error-prone repair (33). These experimental observations are compatible with the strong deletion- and complex-event associations observed here and with their localization near predicted Z-DNA boundaries, although our genomic analyses do not identify the molecular pathway responsible for individual mutations.

A second, non-exclusive explanation involves the broader topological environment in which Z-DNA-forming sequences occur. Transcription generates torsional stress, including negative supercoiling behind transcription complexes, that can promote Z-DNA as well as other non-canonical structures such as R-loops and G-quadruplexes; topoisomerases act to resolve this topological stress through transient DNA cleavage and re-ligation (34). Recent analysis of approximately 6,500 cancer genomes further showed that TOP2B-bound sites are enriched for localized somatic mutations and structural variants, particularly at highly transcribed and chromatin-interacting loci (35). Thus, part of the association between predicted Z-DNA-forming sequences and mutation may arise from their co-localization with transcriptionally active, topologically stressed genomic environments that are themselves susceptible to DNA breakage and repair. This consideration is particularly relevant to the stronger associations observed in genic and euchromatic compartments. Our analyses establish spatial associations with predicted Z-DNA-forming sequences but cannot separate the contributions of Z-DNA formation itself from correlated transcriptional, topological, and repair processes.

The genomic-context dependence observed here is also consistent with recent analyses of short tandem repeat evolution. Goldberg et al. (36) found that predicted Z-DNA formation was associated with STR interruption patterns even after accounting for allele length, GC content, and repeat-unit length, and that these relationships differed according to regulatory context. Such observations reinforce the view that the consequences associated with Z-DNA-forming sequence are not determined by sequence propensity alone but depend on the genomic environment in which the sequence occurs. Similarly, the increased Z-DNA/control ratio among higher-frequency noncoding SNVs in the HPRC data may reflect a combination of mutation, tolerance, and selection rather than any single process.

These findings may also be relevant to disease-associated genomic variation. Previous Z-DNABERT analyses reported overlaps between predicted and experimentally supported Z-forming loci and Mendelian disease-associated variants, including loss-of-function variation (22). In the T2T genome, we additionally observed high predicted Z-DNA density at the SST1 and D4Z4 repeat arrays. SST1 is associated with recurrent Robertsonian translocation breakpoints on acrocentric short arms, whereas contraction of D4Z4 is associated with facioscapulohumeral muscular dystrophy. These colocalizations raise the possibility that Z-DNA-forming potential contributes to the structural properties of these loci, but direct experimental evidence will be required to determine whether Z-DNA formation contributes to their fragility.

Several limitations should therefore be considered when interpreting these results. ZSeeker identifies sequence propensity to form Z-DNA rather than direct Z-DNA occupancy in a particular cell type or physiological state, and structure formation will depend on transcription, supercoiling, chromatin state, replication, and other cellular factors. In addition, although the matched-control analyses account for interval length, GC content, and CpG/GpC dinucleotide density, they do not eliminate every sequence or genomic feature correlated with Z-DNA-forming potential. Finally, the replication of mutation enrichment in de novo variants argues against population-level selection as the sole explanation for the observed association but does not by itself establish a direct causal effect of Z-DNA formation. Future experimental and genomic studies will be required to separate these contributions and determine how Z-DNA formation interacts with replication, transcription, topoisomerase activity, and DNA repair across different genomic contexts.

## Methods

### Prediction of Z-DNA sequences with ZSeeker

Z-DNA sequences were predicted using the ZSeeker software (v1.8) (30) across all 464 human HPRC haplotypes, and the T2T-CHM13, the GRCh38, and the GRCh37 reference genomes. Scoring and penalty settings were left at their default values. ZSeeker outputs non-overlapping Z-DNA predictions per chromosome, and we have used these predictions without reducing overlapping regions to one Z-DNA site.

### Z-DNA density and enrichment

Z-DNA density was defined as the number of ZSeeker-predicted Z-DNA bases per length of the examined region. For Z-DNA enrichment, two different estimations were employed based on the desired question/analysis. First, density enrichment: the Z-DNA base density within a given interval, annotation class, or local window divided by the genome-wide Z-DNA base density. Second, control-referenced enrichment: for each mutation anchor, Z-DNA coverage at positions across a ± 500 bp window was computed and normalized to the window mean, yielding a unitless relative density; the same was applied to the composition- and length-matched control intervals. Enrichment was quantified as the ratio of the Z-DNA to control relative density, or as the discordant odds ratio of mutations uniquely proximal to Z-DNA versus uniquely proximal to control. Window sizes, statistical tests, and multiple-testing corrections are specified per analysis in the corresponding Results sections and/or figure legends.

### Mutational analysis

Variant sites were extracted from the HPRC v2.0 Minigraph-Cactus VCF projected onto T2T-CHM13. We used a custom Python parser that processes the VCF record by record and classifies each reference/alternate allele pair from the two allele lengths in a fixed precedence. Writing L_ref and L_alt for the reference and alternate lengths: a pair with L_ref = L_alt = 1 was a SNV, equal lengths greater than 1 were a MNV when L ≤ 20 bp and a substitution when L > 20 bp, L_ref < L_alt was an insertion when L_ref = 1 and otherwise a complex (insertion-like) event and L_ref > L_alt was a deletion when L_alt = 1 and otherwise a complex (deletion-like) event. Each variant was reduced to a single 1-bp anchor used for all downstream proximity and metaprofile analyses: the variant base for SNVs, the reference-allele midpoint for MNVs and substitutions, the left breakpoint (first changed base) for deletions and deletion-like complex events, and the insertion site for insertions and insertion-like complex events. Multiallelic records were decomposed so that each alternate allele formed an independent entry on its own row (one variant per row).

For each allele, allele count and allele number were recomputed directly from the phased genotypes rather than taken from the INFO fields: missing calls were excluded from AN, and allele frequency was AC/AN within the HPRC sample, termed as graph-internal frequency and not a population-genetic estimate.

The identical classification and anchoring pipeline was applied to *de novo* mutations from denovo-db, Seattle, WA, on the GRCh37/hg19 assembly, restricted to WGS-derived calls (records whose STUDIES SequenceType was "genome"), yielding 255,841 variants; INFO and allele-frequency fields were not used. Because the *de novo* callset is substantially smaller than the population data, variants were not size-stratified but pooled into mutation-class profiles (SNV, MNV, and simple and complex insertions and deletions), of which the multi-nucleotide class (*n* = 481) was excluded for insufficient counts (< 500).

### Generation of the control group

For each predicted Z-DNA interval identified by ZSeeker on the T2T-CHM13, GRCh38 and GRCh37 reference genomes, we generated a composition-matched control sequence on the same chromosome. Control candidates were required to match the Z-DNA interval exactly in length and to fall within a predefined tolerance range for both G + C content and CpG/GpC dinucleotide density (± 10%). Candidate control intervals were sampled at random genomic positions on the same chromosome and screened to ensure that they did not overlap any Z-DNA interval or previously assigned control. To enable efficient evaluation of sequence composition, cumulative base and dinucleotide counts were precomputed for each chromosome. For each Z-DNA interval, control candidates were iteratively sampled and tested until a suitable match was identified or a maximum number of attempts was reached. In cases where random sampling failed, a fallback strategy was applied in which candidate intervals were drawn from available non-overlapping genomic regions on the same chromosome. All accepted control intervals were non-overlapping and composition-matched to their corresponding Z-DNA interval by construction. Using this procedure, composition-matched controls were successfully assigned to 187,749 of 244,989 predicted Z-DNA intervals in GRCh37 (76.6%) and 204,721 of 274,037 intervals in T2T-CHM13 (74.7%). The remaining intervals could not be assigned a suitable control, primarily because no non-overlapping genomic region satisfying the composition-matching criteria could be identified. In analyses, where Z-DNA predictions are compared to the control sequences, only the composition-matched subset was considered.

### Intron inference

Because the reference annotation did not explicitly include intronic regions, they were inferred from exon annotations. Protein-coding genes and their associated transcripts were first identified from the GFF annotation. For each transcript with at least two annotated exons, exon coordinates were converted to 0-based half-open intervals and sorted along the transcript. Introns were then defined as the genomic intervals between consecutive exons within each transcript. All inferred intron coordinates were exported in BED format for downstream analyses.

### Allele-frequency CDS-vs-noncoding analysis

SNV allele counts and allele numbers were taken per allele as described above, and allele frequency was AF = AC/AN within the HPRC sample (a graph-internal frequency, not a population estimate). Alleles were grouped into four frequency bins - AF < 0.25% (singletons at the applied AN floor), 0.25-1%, 1-5%, and > 5% - and divided by genomic compartment: coding-sequence versus noncoding. CDS intervals were taken as the CDS features of the Liftoff v5.2 GFF annotation, converted to 0-based half-open coordinates and merged per chromosome; a SNV anchor falling within a merged CDS interval was assigned to CDS and the rest to noncoding. The primary analysis required AN ≥ 250 and retained singletons, with a minimum of 500 alleles per bin. For each compartment × AF bin, we computed the Z-DNA/control peak ratio: the mutation-anchored metaprofile over ± 500 bp was built separately for Z-DNA and matched control, each normalized to its window mean, and the ratio of mean normalized densities over ± 5 bp of the anchor was taken.

### Statistical analysis

Confidence intervals and significance were estimated using a chromosome-level block bootstrap. Correlations were assessed using Spearman’s ρ, paired comparisons using the Wilcoxon signed-rank test, group differences using the Mann-Whitney U test, and variant proximity to Z-DNA versus matched controls using discordant (McNemar) odds ratios. Multiple-testing correction was performed using the Benjamini-Hochberg procedure, and adjusted p-values are reported as q-values.

## Supplementary Material

This is a list of supplementary files associated with this preprint. Click to download.

SupplementaryMaterial.docx

## Figures and Tables

**Figure 1:**
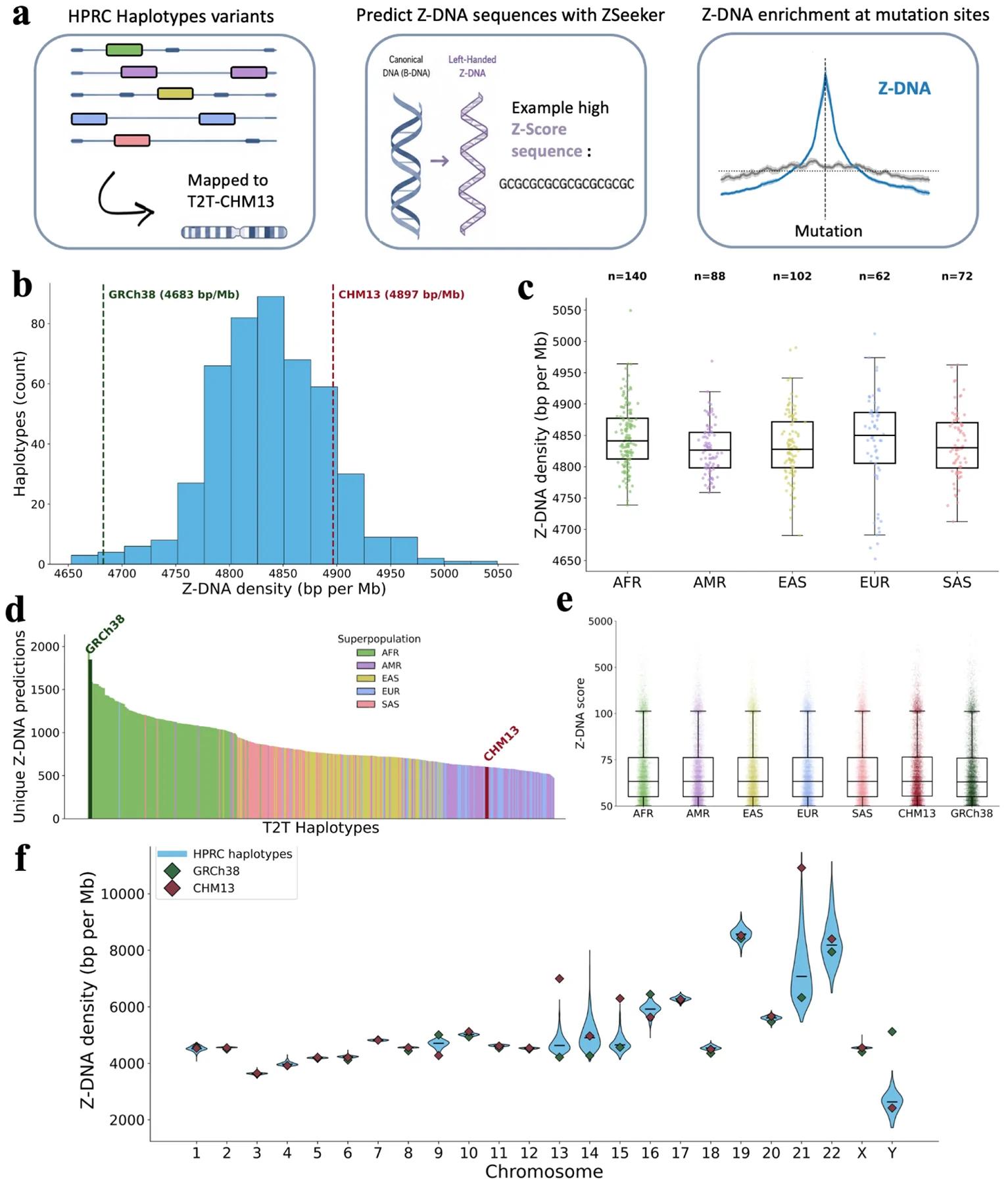
Genome-wide Z-DNA density is broadly similar across human haplotypes. **a.** Simplified schematic illustration of our approach to evaluate Z-DNA density and impact on genetic instability. **b.** Distribution of Z-DNA density (bp/Mb) across 464 HPRC haplotypes and the two human reference genomes; T2T-CHM13 highlighted in dark red and GRCh38 in green. **c.** Violin plots depicting Z-DNA density stratified by superpopulations. **d.** Bar plots showing haplotype-specific Z-DNA sequence count for all 466 genomes, colored by superpopulation and reference genomes. **e.** Box plots of Z-DNA score distributions for the same categories. Each category was randomly sampled to include 200,000 Z-DNA-forming sequences. **f.** Chromosome-level Z-DNA density across haplotypes visualized with violin plots.

**Figure 2:**
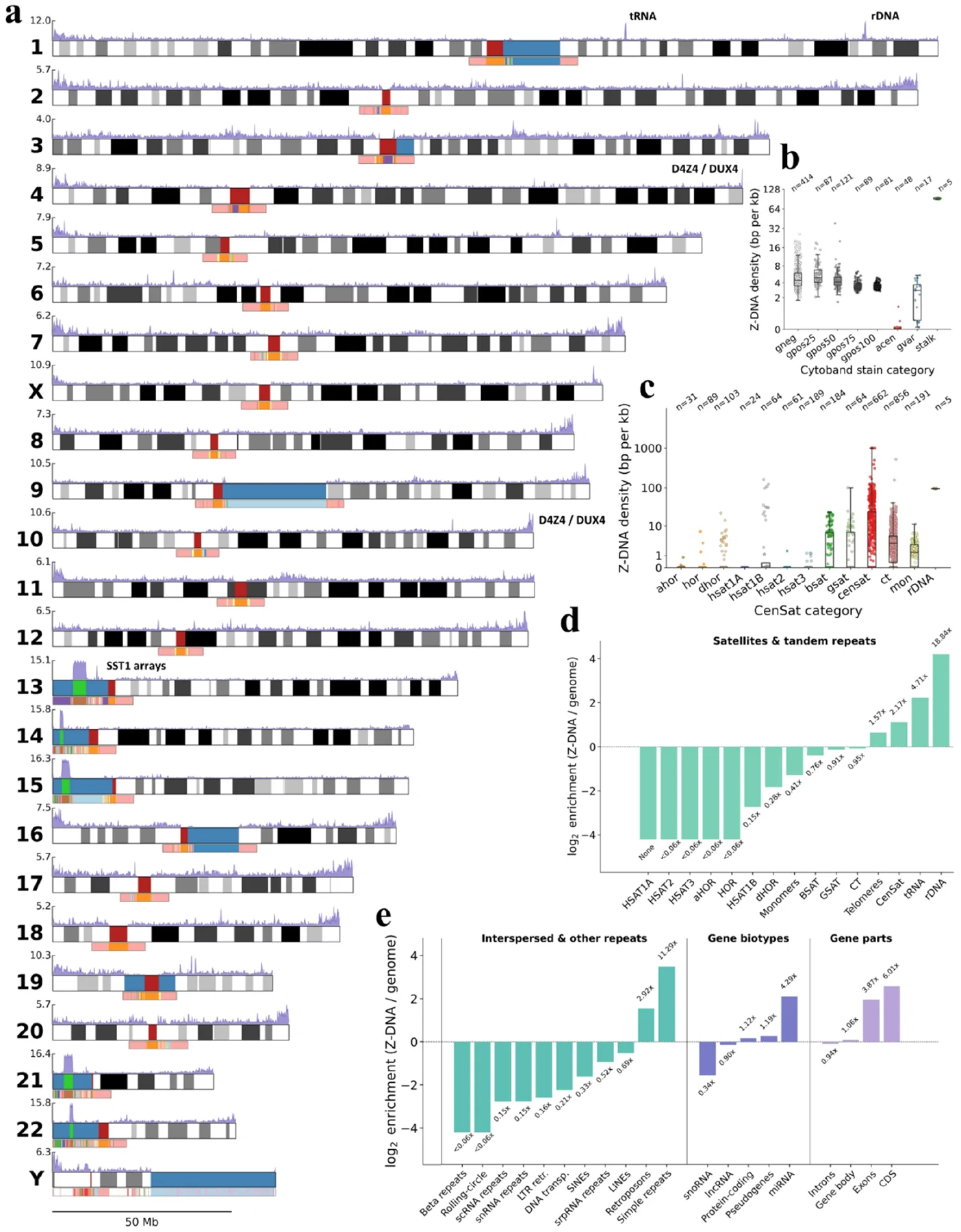
Z-DNA-forming sequences are non-uniformly distributed across the human genome. **a.** Karyoplot of Z-DNA density (purple upper track, 150 kb bins, y-axis featuring Z-DNA density in kb / 150 kb) across all chromosomes of the T2T-CHM13 assembly. Cytobands (middle tracks) are shown with standard Giemsa stain coloring, and CenSat annotation categories are featured in the lower track. Manual text annotations indicate strong local Z-DNA signal. **b.** Z-DNA density (bp per kb, log_2_-scaled y-axis) across cytoband stain categories. Boxplots show median and interquartile range; individual cytoband intervals are overlaid as jitter points. **c.** Z-DNA density (bp per kb, log_10_-scaled y-axis) across CenSat annotation categories, displayed as in b. **d-e.** Log_2_ Z-DNA enrichment relative to genome-wide Z-DNA density across 34 annotation categories: satellites and tandem repeats (d.); interspersed and other repeats; gene biotypes; and gene substructures (e). Fold-change values are labeled above each bar. Repeat abbreviations are presented in **Supplementary Table 2**.

**Figure 3:**
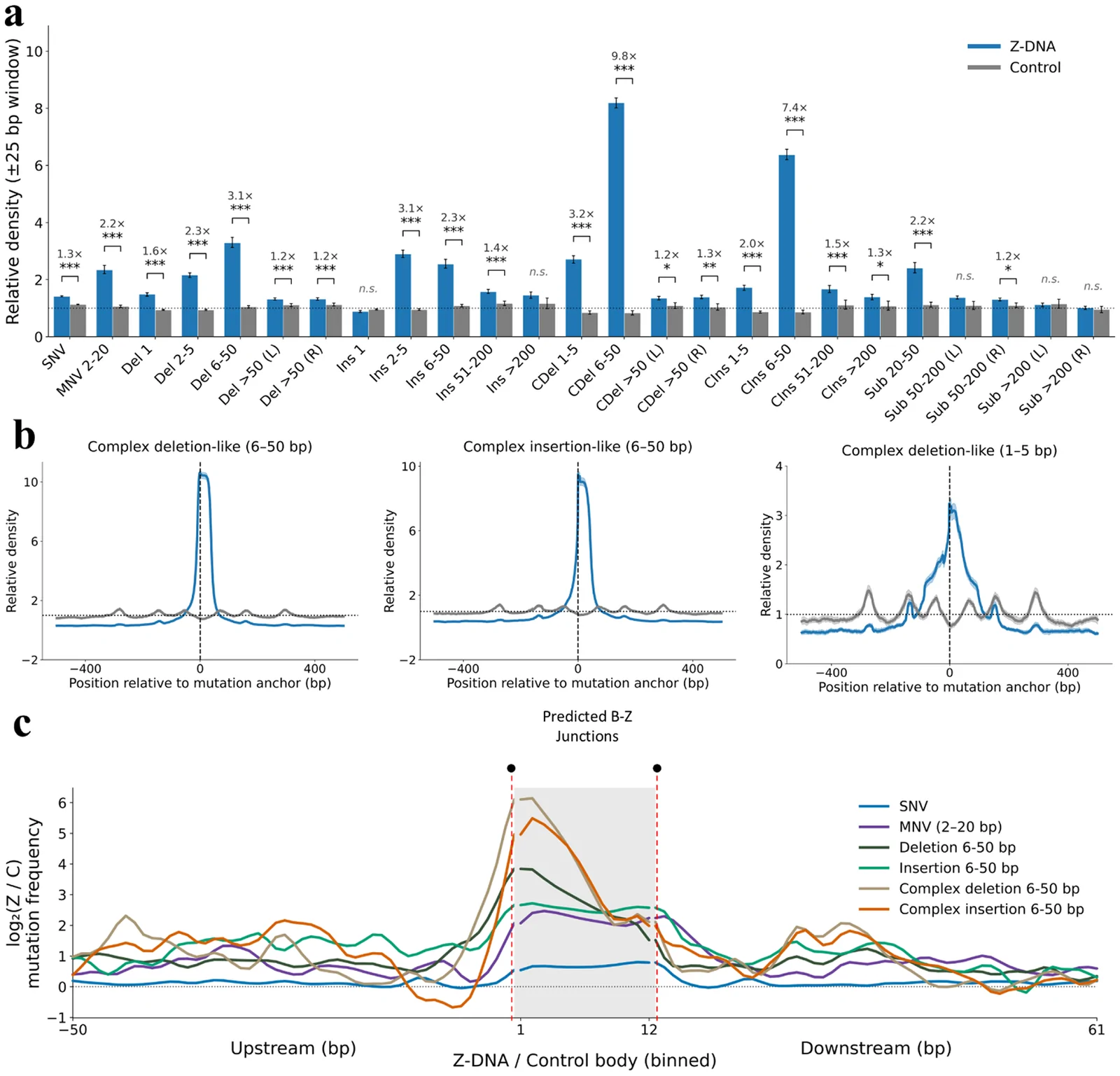
Predicted Z-DNA-forming sequences feature distinct profiles around mutational hotspots. **a.** Bar chart of relative densities of Z-DNA (blue) predictions versus Controls (gray) around a 50 bp window of 21 different mutation categories from the HPRC-CHM13 variant dataset. For dual-anchor strata, both breakpoints are shown (L for left and R for right). Stars denote Wilcoxon signed rank test significance after Benjamini-Hochberg FDR correction, while error bars indicate 95% chromosome block-bootstrap confidence intervals. **b.**Z-DNA metaprofiles for the three most highly Z-DNA-enriched mutation categories from the HPRC-CHM13 variant dataset. For each type, Z-DNA overlap (blue) and matched control overlap (gray) were calculated in a ±500 bp window around the mutation point and normalized over the window mean to give relative density. Shaded bands show 95% chromosome-block bootstrap confidence intervals (CIs). The dashed vertical line marks the mutation position (offset = 0) and the horizontal one indicates the normalized baseline. **c.** Z-DNA-centered metaprofiles for different mutation categories. Mutation frequencies were calculated across 50 bp upstream and downstream regions and across the Z-DNA/control interval body (rescaled into equal bins due to varying Z-DNA lengths). The gray central region represents the Z-DNA/control body.

**Figure 4:**
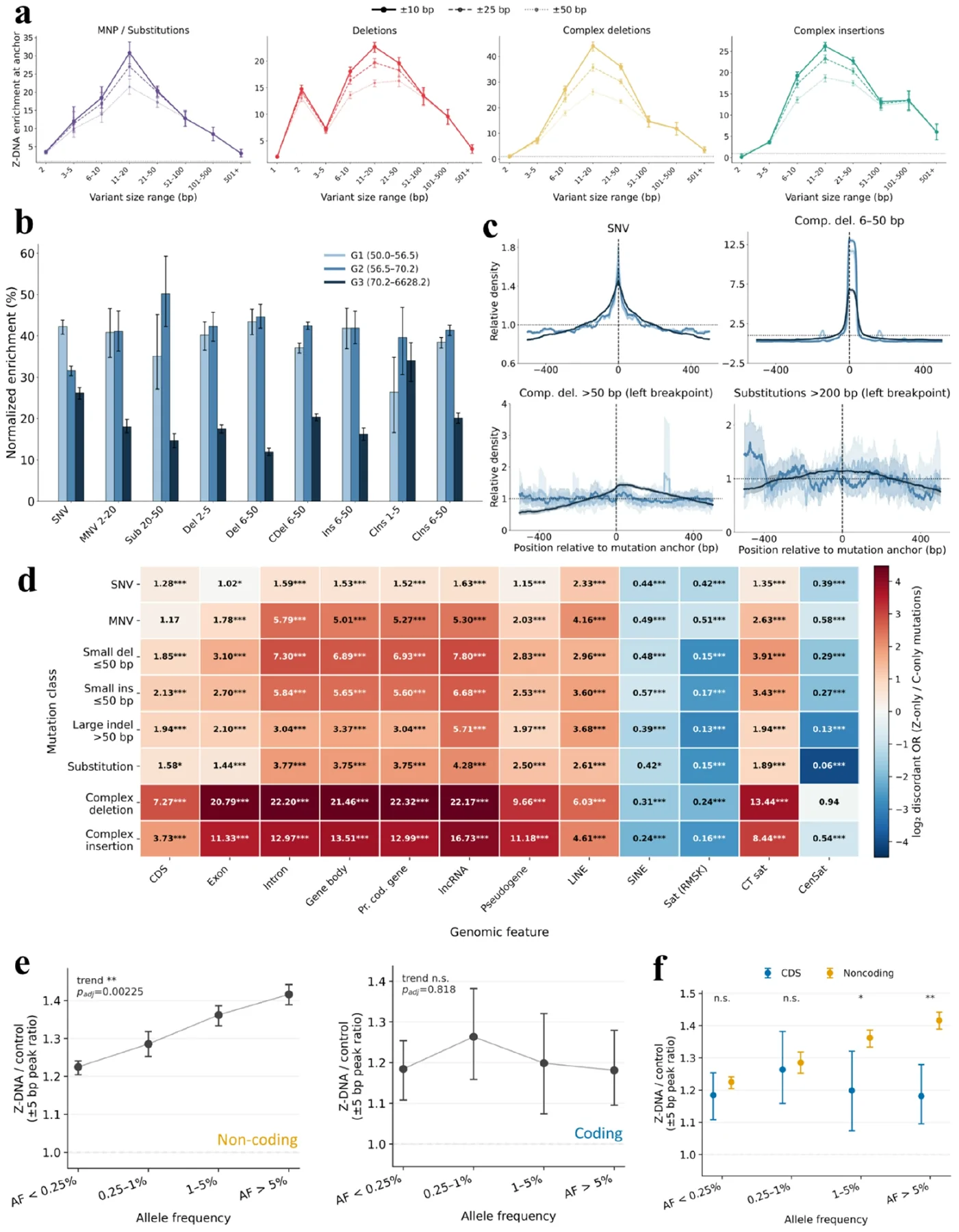
Z-DNA-associated mutagenesis depends on variant size, Z-tract score, localization and allele frequency. **a.** Z-DNA enrichment as a function of variant size. Enrichment is the ratio of the local Z-DNA base fraction (within ±10, ±25, or ±50 bp of the mutation anchor) to the genome-wide Z-DNA fraction (0.48%). Points: chromosome-block bootstrap means; error bars: 95% CIs. **b.**Score-stratified Z-DNA enrichment for representative mutation strata. Predicted Z-DNA regions were divided into three equal-count groups by Z-DNA score: G1 (50.0-56.5, light blue), G2 (56.5-70.2, medium blue), and G3 (70.2-6628.2, dark blue). Bars show each score group’s contribution to the total excess Z-DNA-associated signal within each mutation stratum, normalized to 100% across G1-G3. Error bars indicate 95% chromosome-block bootstrap confidence intervals. Pairwise differences between score groups were assessed using chromosome-level Wilcoxon signed-rank tests with BH-FDR correction. **c.** Score-stratified mutation-anchored metaprofiles for SNVs, complex deletions 6-50 bp, complex deletions >50 bp, and substitutions >200 bp. For dual-anchor classes, the left breakpoint is shown. Lines show self-normalized relative Z-DNA density across ±500 bp from the mutation anchor for each Z-DNA score group; shaded bands indicate 95% chromosome-block bootstrap confidence intervals. The dashed vertical line marks the mutation anchor and the dotted horizontal line marks the normalization baseline. **d.** Discordant odds ratio heatmap (variants uniquely proximal to Z-DNA vs. uniquely proximal to matched control) for each mutation class across genomic features. Anchors within ±10 bp of a Z-DNA interval and of a length-/composition-matched control interval were scored per variant; the McNemar OR contrasts the two. Color: log_2_ OR (red, enriched near Z-DNA; blue, depleted). Stars indicate BH-FDR-adjusted significance (*: *q* < 0.05, **: *q* < 0.01, ***: q < 0.001). Features overlap, so ORs are not additive across columns. Features failing the Z/control matching were excluded. **e.** Z-DNA/control density ratio at SNV sites (±5 bp metaprofile peak) as a function of within-HPRC allele frequency, in noncoding (left) and coding (right) sequences. Points: chromosome-block bootstrap means; error bars: 95% CIs. Trend q: BH-FDR-adjusted bootstrap test of the slope across AF bins. **f.** Direct comparison of the ±5 bp Z-DNA/control peak ratio between CDS (blue) and noncoding (orange) per allele-frequency bin. Error bars: 95% bootstrap CIs. Stars: BH-adjusted between-region test (*n.s.*: not significant, *: *q* < 0.05, **: *q* < 0.01).

**Figure 5:**
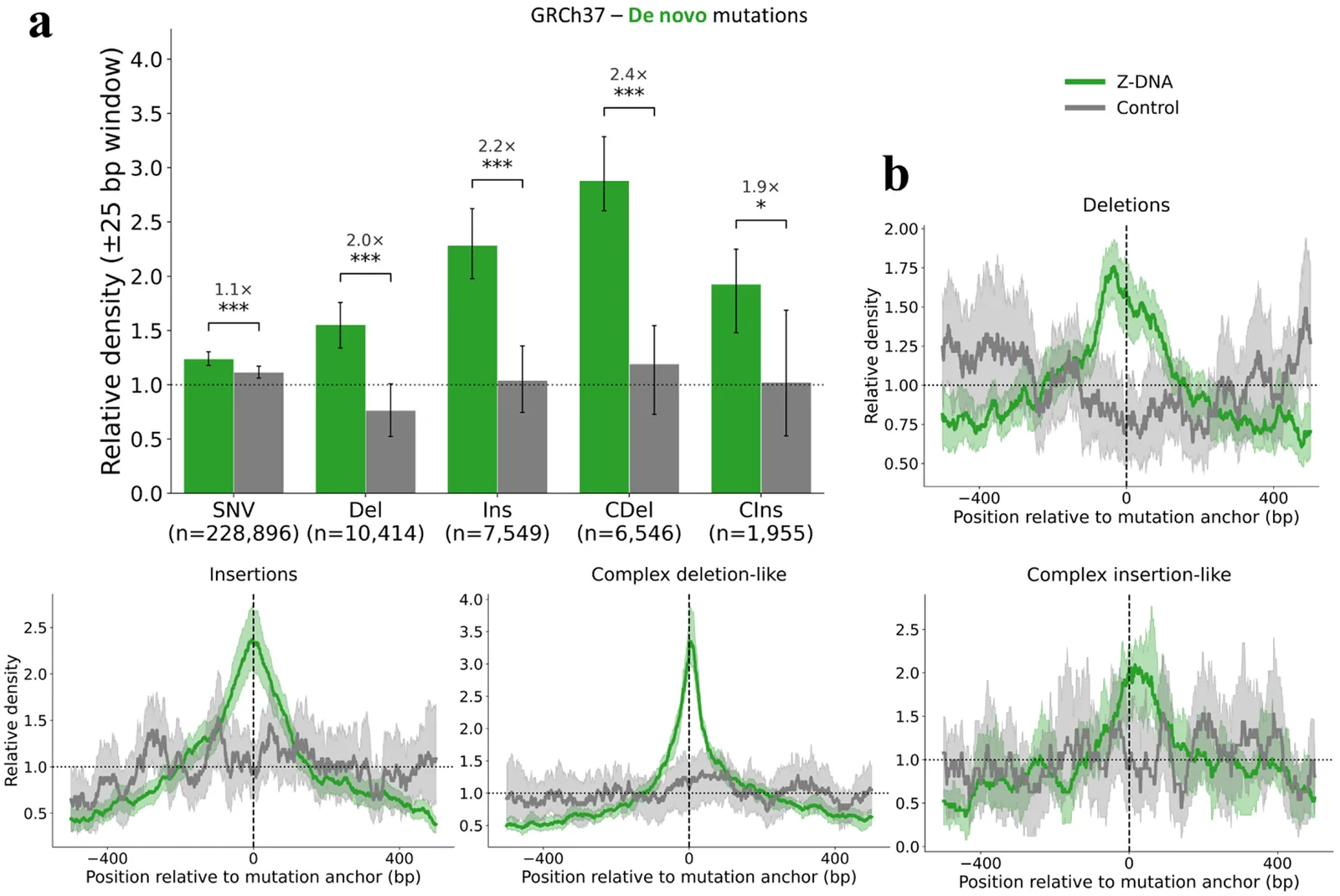
*De novo* mutations are enriched at predicted Z-DNA loci. **a.**Relative density of *de novo* mutations within ±25 bp of predicted Z-DNA regions (green) versus composition-matched control regions (grey), grouped by mutation class. Bars show the window-averaged relative density (self-normalized to the ±500 bp profile mean); error bars indicate 95% chromosome block-bootstrap confidence intervals. Fold enrichment and significance are annotated from one-sided chromosome-block bootstrap tests after BH-FDR correction (*: *q* < 0.05, **: *q* < 0.01, ***: *q* < 0.001). Sample sizes are shown below each class. MNVs (n = 481) were excluded due to insufficient counts (<500). **b.** Per-class relative-density metaprofiles across the ±500 bp window centered on the mutation anchor. Shaded bands represent 95% chromosome block-bootstrap confidence intervals. The dashed vertical line marks the mutation site; the dotted horizontal line marks the self-normalized baseline.
